# Comparative Transcriptome Analysis to Reveal Genes Involved in Wheat Hybrid Necrosis

**DOI:** 10.3390/ijms151223332

**Published:** 2014-12-16

**Authors:** Yong Zhang, Yan Cheng, Jiahui Guo, Ennian Yang, Cheng Liu, Xuelian Zheng, Kejun Deng, Jianping Zhou

**Affiliations:** 1 School of Life Science and Technology, University of Electronic Science and Technology of China, Chengdu 610054, China; E-Mails: zhangyong916@uestc.edu.cn (Y.Z.); cy505518162@163.com (Y.C.); guojiahui@uestc.edu.cn (J.G.); zhengxl@uestc.edu.cn (X.Z.); dengkj@uestc.edu.cn (K.D.); 2 Crop Research Institute, Sichuan Academy of Agricultural Sciences, Chengdu 610066, China; E-Mail: yangennian@126.com; 3 Crop Research Institute, Shandong Academy of Agricultural Sciences, Ji’nan 250100, China; E-Mail: liucheng@uestc.edu.cn

**Keywords:** *Triticumaestivum*, hybrid necrosis, digital gene expression, transcriptome

## Abstract

Wheat hybrid necrosis is an interesting genetic phenomenon that is found frequently and results in gradual death or loss of productivity of wheat. However, the molecular basis and mechanisms of this genetic phenomenon are still not well understood. In this study, the transcriptomes of wheat hybrid necrosis F_1_ and its parents (Neimai 8 and II469) were investigated using digital gene expression (DGE). A total of 1300 differentially expressed genes were identified, indicating that the response to hybrid necrosis in wheat is complicated. The assignments of the annotated genes based on Gene Ontology (GO) revealed that most of the up-regulated genes belong to “universal stress related”, “DNA/RNA binding”, “protein degradation” functional groups, while the down-regulated genes belong to “carbohydrate metabolism” and “translation regulation” functional groups. These findings suggest that these pathways were affected by hybrid necrosis. Our results provide preliminarily new insight into the underlying molecular mechanisms of hybrid necrosis and will help to identify important candidate genes involved in wheat hybrid necrosis.

## 1. Introduction

Hybrid necrosis (sometimes known as hybrid weakness) is associated with characteristic phenotypes that include cell death, tissue necrosis, wilting, yellowing, chlorosis, dwarfism, and reduced growth rate, and often results in lethality [1]. Hybrid necrosis is a serious barrier either to desirable trait combinations from different genotype cultivars or gene transference from related species to commercial cultivars [2,3]. Hybrid necrosis belongs to postzygotic hybrid incompatibilities that involve epistatic interactions as predicted by the Bateson–Dobzhansky–Muller (BDM) model [4]. Studies further demonstrated that hybrid incompatibility relies on activation of the salicylic acid (SA) stress signaling pathway [5]. Moreover, it is hypothesized that hybrid necrosis can result from autoimmunity, a pleiotropic effect of evolutionary genes that are involved in the pathogen response [1]. Dalal and Khanna-Chopra *et al.* [6,7] reported that hybrid necrosis in wheat leaves was associated with oxidative stress resulting from a not well-coordinated antioxidant defense system. However, detailed molecular mechanisms associated with hybrid necrosis are still not well understood.

Common wheat (*Triticumaestivum* L. AABBDD, 2n = 42), one of the most important food crops in the world, occupies 17% of all the cultivated land and accounts for 20 percent of the calories consumed by humans [8,9]. Hybrid necrosis has been frequently observed in F_1_ hybrids between genotypes of common wheat [10], which is usually lethal or semi-lethal, resulting in its gradual death or loss of productivity [2,11,12]. Although hybrid necrosis in wheat was first reported in the 1940’s [13] and a series of classical research studies revealed that this phenomenon is genetically controlled by two complementary dominant genes *Ne1* and *Ne2* located on chromosome arms 5BL and 2BS, respectively [12,14,15,16,17], the molecular mechanisms associated with hybrid necrosis in wheat are still not understood.

Transcriptome sequencing using next generation sequencing technology to provide high-resolution data is a powerful tool for studying global transcriptional networks. The evaluation of sequence-based expression profiles can identify stress responsive genes and provide genes with functional annotation. Recently, transcriptome sequencing has not only been used in model plants [18,19,20], but also in non-model plants whose genomes have never been sequenced [21,22,23,24,25]. In wheat, transcriptome analysis has been used to study grain protein content related genes [26], the polyploidization events [27], and expression profiles in responses to abiotic stress, such as H_2_O_2_ treatments [28], Pi starvation [29] and cold treatments [30]. In the present study, we sampled the pooled transcriptomes of wheat hybrid F_1_ (dwarfness) and its control (its parents) using Illumina paired-end sequencing technology to generate a large-scale expressed sequence tag (EST) database. The assembled and annotated gene expression profiles will provide a valuable resource to identify differentially expressed genes during hybrid necrosis, and will enable us to understand the underlying molecular mechanism of hybrid necrosis. The EST datasets together with the new transcript data will also serve as a good resource for novel gene discovery and marker-assisted selection in wheat breeding.

## 2. Results

### 2.1. Illumina Sequencing and Gene Annotation

**Figure 1:**
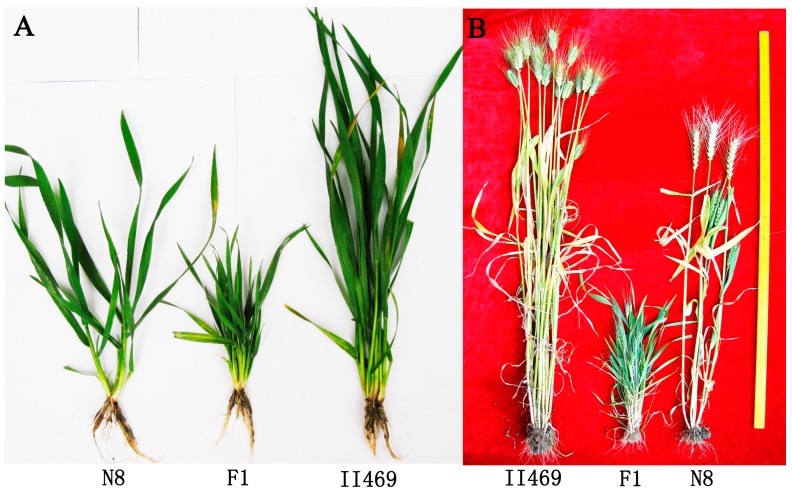
The phenotype of wheat hybrid necrosis F_1_ hybrids and its parents, Neimai8 (N8) and II469 at the seedling stage (**A**) and at the harvest stage (**B**).

The F_1_ hybrids between common wheat Neimai8 (N8) and II469 show hybrid dwarfness belongs to necrosis (Figure 1). To obtain a comprehensive survey on genes related to wheat hybrid necrosis, three libraries (F_1_, N8 and II469) were constructed for sequencing (Table 1). More than 80 million original sequencing tags were produced, representing 38,517,039 and 24,465,242 and 21,801,556 raw reads from the library of F_1_, N8 and II469, respectively. And the percent of A, T, G, C was approximately equal and the GC content of raw reads was about 54%. After trimming the low-quality reads (low quality tags, tags containing N and tags of low quality), more than 33.97 million, 21.66 million and 19.21 million clean reads were obtained from the library of F_1_, N8 and II469, respectively. 55.9%, 55.5% and 52.8% of these clean reads from the library of F_1_, N8 and II469, respectively, were mapped perfectly onto the reference sequences [8] for a total of 111,328 unigenes which were aligned with the Nr, Swiss-Prot, the KEGG and COG database using BLASTx (Supplementary Data 1: Table S1).

**Table 1:** Statistics of trimmed reads and percent of mapping reads.

Sample	Raw Reads	High Quality Pair Reads	High Quality Single Reads	High Quality Nucleotides (bp)	Percent of Mapping Reads
F_1_	38,517,039 × 2	24,611,127 × 2	9,366,249	5,682,981,856	55.87%
II469	21,801,556 × 2	13,911,571 × 2	5,306,501	3,206,968,477	55.50%
N8	24,465,242 × 2	15,731,640 × 2	5,929,060	3,626,936,717	52.83%

### 2.2. Changes in Global Gene Transcription under Hybrid Necrosis

To characterize the genes involved in hybrid necrosis, the expression profiles of F_1_ were compared with its parents (N8 and II469). A statistical analysis of the frequency of genes identified 1300 differentially expressed genes under hybrid necrosis (Supplementary Data 2: Table S2).

An annotation analysis revealed that nearly 40% (38.5%, 501/1300) of the differentially expressed genes were “functional unknown”, annotated as “uncharacterized”, “hypothetical protein”, “predicted protein” or “not found”. Moreover, 360 genes, accounting for 27.7% of all differentially expressed genes, did not match to known sequences, defined as “not found”, which suggested our study may allow us to identify novel genes involved in hybrid necrosis.

**Figure 2:**
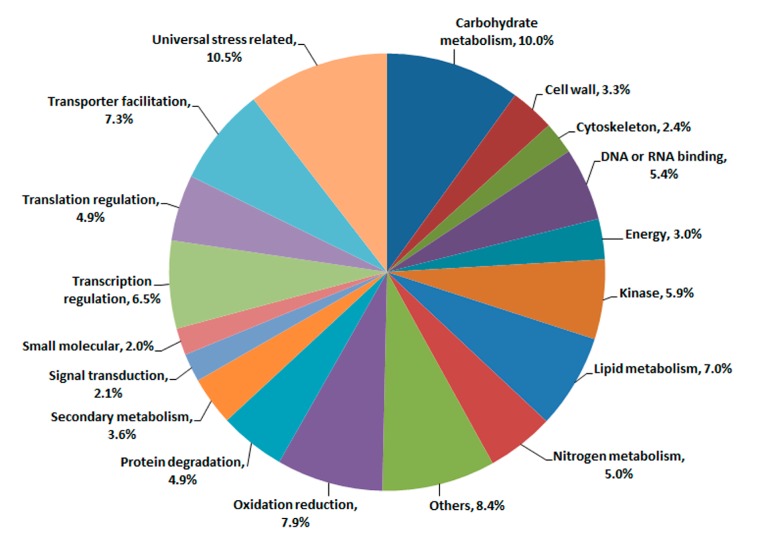
Functional categorization of all annotated and differentially expressed genes. This analysis was based on annotated genes, not including genes classified as “unknown function”.

Based on GO analysis results, 799 annotated genes were categorized into 18 functional categories (Figure 2). The largest categories were “universal stress related” (10.5%) and “carbohydrate metabolism” (10.0%). The expected group associated with “universal stress related” represented for 84 genes. And in the group of “universal stress related”, genes related to “disease resistance, pathogenesis-related and defense genes” (18 genes) and “heat/cold shock protein, temperature responsive genes” (17 genes) were also identified. As expected, 58 genes (7.3%) were categorized into the group of “transporter facilitation”. We also found a high percentage of genes related to “oxidation reduction” (63 genes) and “lipid metabolism” (56 genes). In addition, 6.5% genes identified were related to “transcription regulation”, and 4.9% genes expressed differentially were involved in “translation regulation”. Interestingly, 43 genes (5.4%) involved in “DNA or RNA binding” were found to be differentially expressed under hybrid necrosis. Additionally, the categories related to “kinase” and “nitrogen metabolism” had 47 genes (5.9%) and 40 genes (5.0%) identified, respectively. A total of 39 genes (4.9%) and 29 genes (3.6%) were involved in “protein degradation” and “secondary metabolism”, respectively. A substantial number of genes were also found in “cell wall” (3.3%) and “energy” (3.0%) functional groups. Nineteen genes were involved in “cytoskeleton” and 17 genes related to “signal transduction” were also identified. At the same time, 16 genes involved in “small molecular” were also found. Many significantly changed genes (8.4%) involved in a variety of pathways were affected by hybrid necrosis and were categorized into the group of “other function”.

### 2.3. Transcriptomic Comparison of F_1_ and Its Parents Using Digital Gene Expression (DGE) Profiling

**Figure 3:**
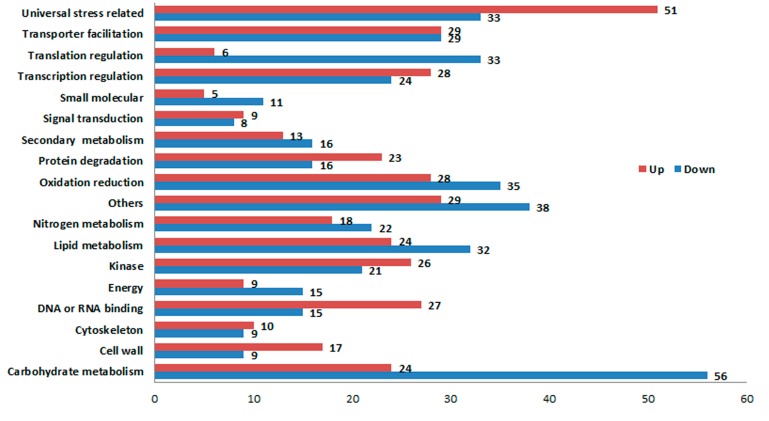
Comparison between up-regulated and down-regulated genes based on functional categories.

**Figure 4:**
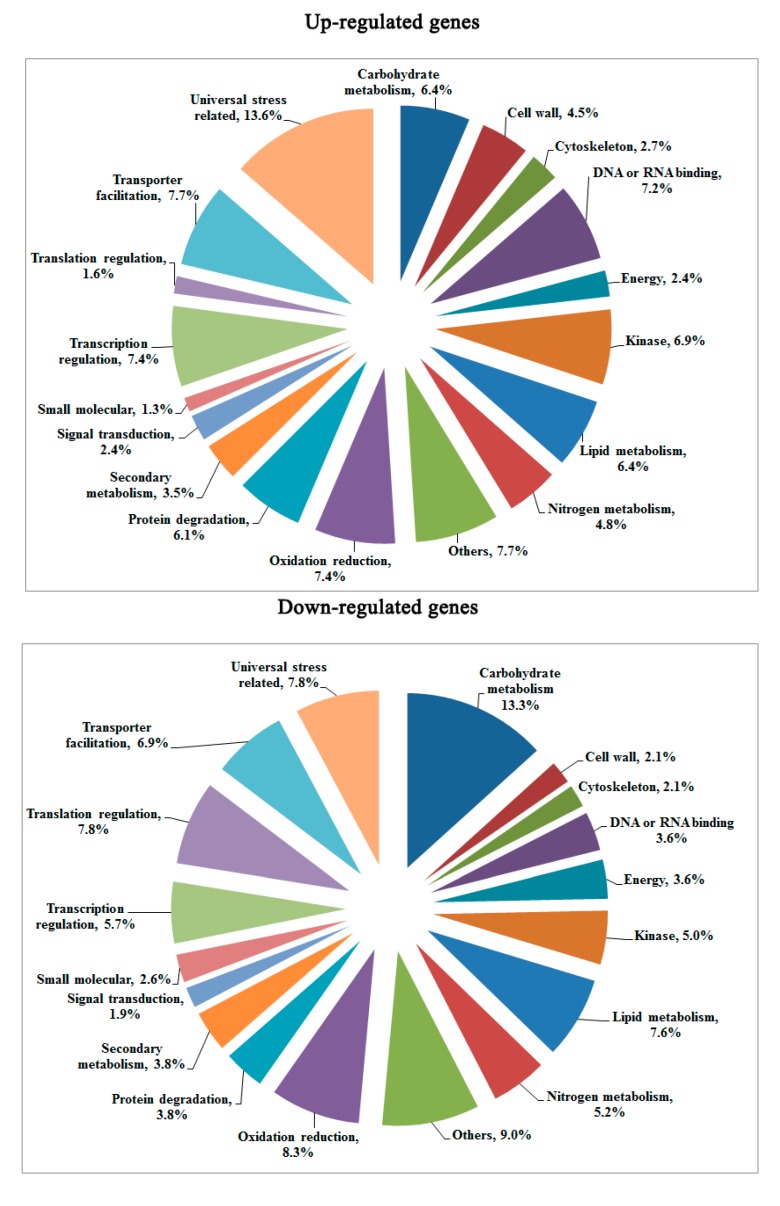
Functional categorization of up-regulated and down-regulated genes. This analysis did not include the genes with “unknown function”.

The expression profiles of F_1_ compared to its parents (N8 and II469). Of the1300 differentially expressed genes, 637 genes were up-regulated and 663 genes were down-regulated. Based on the categorization of up-regulated and down-regulated genes respectively, a comparison between these categories was performed (Figure 3). As shown in Figure 3, there were more down-regulated genes than up-regulated ones in most categories. There were more down-regulated genes in two categories: “carbohydrate metabolism” and “translation regulation”. However, there were more up-regulated genes in the categories of “universal stress related” and “DNA or RNA binding”. Additionally, 241 genes classified as “unknown function” were found down-regulated, whereas there were up to 260 novel genes up-regulated in hybrid necrosis. To provide further valuable information, Gene Ontology (GO) assignments were performed according to the annotated genes that were up-regulated or down-regulated, respectively (Figure 4). The top three largest groups of up-regulated genes were “universal stress related”, “DNA/RNA binding”, and “protein degradation”, while “carbohydrate metabolism”, “translation regulation”, and “others” were the top three largest groups of down-regulated genes. Surprisingly, genes related to “universal stress related” comprised up to 13.6% of the up-regulated genes.

### 2.4. Validation of DGE Results Using Quantatitive RT-PCR (qRT-PCR)

**Figure 5:**
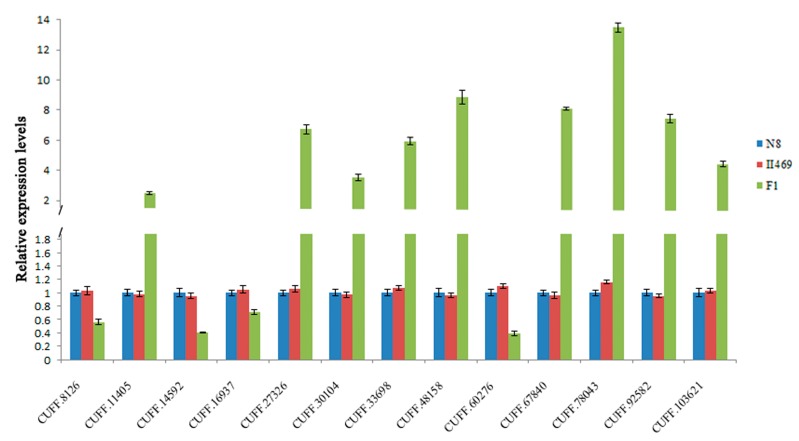
Genes with expression levels validated by qRT-PCR. CUFF.8126: Abscisic stress-ripening protein 2, CUFF.11405: vernalization insensitive 3, CUFF.14592: Defensin-like protein, CUFF.16937: Programmed cell death protein 4, CUFF.27326: senescence-associated-like protein, CUFF.30104: E3 ubiquitin ligase BIG BROTHER-related protein, CUFF.33698: Minor histocompatibilityantigen H13-like, CUFF.48158: BONZAI 3-like, CUFF.60276: auxin transport protein BIG-like, CUFF.67840: Autoimmune regulator, histone H3 acetyltransferaseIDM1, CUFF.78043: Ubiquitin carboxyl-terminal hydrolase 25, CUFF.92582: Histone acetyltransferase HAC5, CUFF.103621: TIFY 10A-like. Bars show standard error.

To validate the results of the DGE data, the transcriptional level of 13 genes selected were examined by qRT-PCR. The annotation information about these genes was listed in Supplementary Data 3: Table S3. As shown in Figure 5, the expression patterns of all selected genes agreed well with the DGE data although the change fold did not exactly match the number revealed by the DGE data for these genes. Interestingly, the autoimmune related gene (CUFF.67840) and protein degradation related gene (CUFF.78043) were up-regulated significantly while auxin transport (CUFF.60276) and defensin-like gene (CUFF.14592) were down-regulated dramatically in F_1_, providing a hint concerning the underlying molecular mechanism of hybrid necrosis.

## 3. Discussion

Digital gene expression by high-throughput sequencing is widely used for transcriptomic analyses. In this study, we applied this method to evaluate gene expression under wheat hybrid necrosis. As expected, many genes were differentially expressed in hybrid F_1_ compared to it parents; some of these genes were further confirmed by qRT-PCR experiments to demonstrate the validity of the DGE data. The GO analysis revealed that these differentially expressed genes were distributed among various pathways, suggesting that a variety of physiological processes were affected by hybrid necrosis.

It was reported that the pathogen effectors trigger the plant’s pathogen-response signal result in hybrid necrosis [31,32,33], Therefore, it is not surprising that the most differentially expressed genes were found to be related to “universal stress related” in F_1_ compared to its parents (Figure 3 and Figure 4). Fourteen of these genes were disease resistance/pathogenesis related genes, for example UFF.13537, CUFF.57510, CUFF.64116, *etc.* (Table S3). Although more disease resistance/pathogenesis related genes were up-regulated, there were some disease resistance/pathogenesis related genes down-regulated, such as CUFF.14592 (defensin-like gene) and CUFF.25645 (disease resistance gene) (Figure 5, Table S1), indicating the diversity of R-genes. Moreover, the previous study showed that BON1 was a negative regulator of a haplotype-specific Resistance (R) gene SNC1 [34]. In the present study, CUFF.48158 (BONZAI 3-like), a member of BON family was obviously up-regulated (Figure 5). And a senescence-associated-like gene, CUFF.27326 was also up-regulated (Figure 5). Accordingly, it was believed that the “universal stress related” genes were involved in hybrid necrosis. In addition, the second largest groups of up-regulated genes were “DNA/RNA binding” in this study (Figure 3), however, this category contains a considerable number of down-regulated genes, such as the gene CUFF.16937 (programmed cell death protein 4) which contains four MA3 domains and has been implicated in ethylene signaling and abiotic stress responses [35] and down-regulated gene CUFF.34538 (protection of telomeres 1) which protect telomeres from degradation [36,37], indicating that the chromosomes of cells might be unusually degraded and that cells died unexpectedly in F_1_, which might be one reason for hybrid necrosis.

Metabolism of carbohydrate, nitrogen and lipid are fundamental and crucial for organisms to maintain normal physiological function [38]. In the present study, more down-regulated genes related to the categories of “carbohydrate metabolism”, “nitrogen metabolism”, “lipid metabolism” and “secondary metabolism”, especially the genes involved in “carbohydrate metabolism” comprised up to 13.6% of the down-regulated genes in F_1_ (Figure 3 and Figure 4), suggesting the different decrease and consumption of carbohydrate, amino acid and fatty acid, which would consequently affect the composition of the body, and finally cause the defects of growth and development in F_1_that result in dwarfness.

The ubiquitin-proteasome pathway is responsible for the major portion of specific cellular protein degradation [39]. Ubiquitin-mediated degradation is involved in physiological regulation of many cellular processes, including cell cycle progression, differentiation, and signal transduction [39]. In the present study, more up-regulated genes were categorized in “protein degradation” (Figure 3 and Figure 4), such as CUFF.30104 (E3 ubiquitin ligase BIG BROTHER-related gene) which is involved in multiple plant developmental processes [40] and plant response to abiotic stress [41,42], and CUFF.78043 (ubiquitin carboxyl-terminal hydrolase 25) which has a specific role in development [43], and CUFF.103621 (TIFY 10A-like) which plays important roles in plant environmental stress responses and adaptation [44]. These results imply that the expression changes of ubiquitin-mediated degradation genes for response to hybrid necrosis

In addition, it was proposed that autoimmunity caused hybrid necrosis [1]. In the present research, the immune related genes, CUFF.67840 (autoimmune regulator) [45], CUFF.33698 (minor histocompatibility antigen H13-like) [46] and CUFF.92582 (histone acetyltransferase HAC5) [47] were up-regulated dramatically (Figure 5), supporting the concept that hybrid necrosis can result from autoimmunity.

## 4. Experimental Section

### 4.1. Plant Materials

Hexaploid wheat (*Triticumaestivum* L.) cultivar Neimai8 (N8), line II469 and their (cross and reciprocal cross) F_1_ hybrids were grown in a growth chamber at a relative humidity of 75% and 26/20 °C day and night temperature. Two weeks later, the whole seedling plants including leaves and roots were frozen immediately in liquid nitrogen for RNA extraction. The samples were replicated twice and each sample pool for the transcriptome sequencing was made up of 30 individual seedling plants.

### 4.2. RNA Extraction

Total RNA was isolated using TRIzol^®^ reagent according to the manufacturer’s instructions (Invitrogen, Carlsbad, CA, USA) followed by RNase-free DNase treatment (Takara, Dalian, China). RNA quantity and quality were assessed by a Nanodrop spectrophotometer and by agarose gel electrophoresis.

### 4.3. cDNA Library Development and Sequencing

cDNA libraries were prepared and sequenced according to the manufacturer’s instructions and sequenced on an Illumina HiSeq2000 system (MajorbioBioTech Co., Ltd., Shanghai, China).

### 4.4. Data Filtering and Gene Annotation

For the raw data, the raw reads were filtered and cleaned by removing the adapter sequences, low-quality sequences, tags with unknown nucleotides N, empty reads and tags that were too short or too long to get clean reads using SeqPrep program (MajorbioBioTech Co., Ltd.) and condetri_v2.0.pl program [48].

For Gene annotation, all the clean tags were mapped onto the reference sequences [8] using Tophat program [49] to get unigenes which were aligned with the Nr, Swiss-Prot, the KEGG and COG database using BLASTx with an *E*-value of less than 1 × 10^−5^.

### 4.5. Identification of Differentially Expressed Genes

The expression level of each gene was normalized to RPKM (Reads Per Kb per Million reads) based on the number of clean tags. Genes were deemed significantly differentially expressed using Tophat program [49] and Cuffdiff program [50] with a *p*-value <0.005, FDR < 0.05.

### 4.6. Quantitative Real-Time PCR Analysis

Three biological replications with two technique replications of total RNA were used for quantitative real-time PCR analysis. Total RNA was treated with RNase-free DNase. Reverse transcription reaction of total RNA was performed with a RevertAid First Strand cDNA Synthesis Kit (Thermo Scientific, Waltham, MA, USA), and qRT-PCR was performed with an Super Real PreMix Plus (SYBR Green) PCR master mix kit (Tiangen, Beijing, China) according to the manufacturer’s instructions using a CFX96 Real-Time System C1000 Thermal Cycler (Bio-RAD, Hercules, CA, USA). The primers were listed in Table S3. And the expression of actin was used as an internal control. Values were obtained by normalizing to Actin and then comparing the normalized values to those of control plants. The relative levels of gene expression were calculated using the 2^−ΔΔ*C*t^ method.

## 5. Conclusions

In summary, we used transcript to me analysis to profile differential gene expression underlining the hybrid necrosis in wheat. And over a thousand genes are differentially expressed in the hybrid necrosis F1 wheat plants when comparing the both parents. Among these genes, stress-responsive genes are preferentially induced while many genes in primary metabolism are down-regulated. Although a large number of differentially expressed genes always leads to difficulty in the characterization of the genes that are actually related to hybrid necrosis. Based on our analysis, the genes related to “universal stress related” might be good choice for further study. The major finding in our study provides a good starting point for future functional studies.

## Supplementary Materials

Supplementary tables can be found at http://www.mdpi.com/1422-0067/15/12/23332/s1.

## References

[1] Bomblies K., Weigel D. (2007). Hybrid necrosis: Autoimmunity as a potential gene-flow barrier in plant species. Nat. Rev. Genet..

[2] Tomar S.M.S., Kochumadhavan M., Nambisan P.N.N. (1991). Hybrid weakness in *Triticumdicoccum* Schubl. Wheat Inf. Serv..

[3] Bizimungu B., Collin J., Comeau A., St-Pierre C.A. (1998). Hybrid necrosis as a barrier to gene transfer in hexaploid winter wheat triticale crosses. Can. J. Plant Sci..

[4] Orr H.A. (1996). Dobzhansky, Bateson, and the genetics of speciation. Genetics.

[5] Alcázar R., García A.V., Parker J.E., Reymond M. (2009). Incremental steps toward incompatibility revealed by *Arabidopsis*epistatic interactions modulating salicylic acid pathway activation. Proc. Natl. Acad. Sci. USA.

[6] Dalal M., Khanna-Chopra R. (1999). Lipid peroxidation is an early event in necrosis of wheat hybrid. Biochem. Biophys. Res. Commun..

[7] Dalal M., Khanna-Chopra R. (2001). Differential response of antioxidant enzymes in leaves of necrotic wheat hybrids and their parents. Physiol. Plant..

[8] Brenchley R., Spannagl M., Pfeifer M., bBarker G.L., D’Amore R., Allen A.M., McKenzie N., Kramer M., Kerhornou A., Bolser D. (2012). Analysis of the bread wheat genome using whole-genome shotgun sequencing. Nature.

[9] Gill B.S., Appels R., Botha-Oberholster A.M., Buell C.R., Bennetzen J.L., Chalhoub B., Chumley F., Dvorak J., Iwanaga M., Keller B. (2004). Aworkshop report on wheatgenome sequencing: Interna-Tional Genome Researchon Wheat Consortium. Genetics.

[10] Tsunewaki K. (1992). Aneuploid analysis of hybrid necrosis and hybrid chlorosis in tetraploidwheats using the d-genome chromosome substitution lines of durum wheat. Genome.

[11] Tomar S.M.S., Singh B. (1998). Hybrid chlorosis in wheat × ryecrosses. Euphytica.

[12] Chu C.G., Faris J.D., Friesen T.L., Xu S.S. (2006). Molecular mapping of hybrid necrosis genes *Ne1* and *Ne2* in hexaploid wheat using microsatellite markers. Theor. Appl. Genet..

[13] Caldwell R.M., Compton L.E. (1943). Complementary lethal genes in wheat causing a progressive lethal necrosis of seedlings. J. Hered..

[14] Hermsen J.G.T. (1967). Hybrid dwarfness in wheat. Euphytica.

[15] Nishikawa K., Mori T., Takami N., Furuta Y. (1974). Mapping of progressive necrosis gene *Ne1* and *Ne2* of common wheat by the telocentric method. Jpn. J. Breed..

[16] Singh S., Chaudhary H.K., Sethi G.S. (2000). Distribution and allelic expressivity of genes for hybrid necrosis in some elite winter and spring wheat ecotypes. Euphytica.

[17] Zeven A.C. (1972). Determination of the chromosome and its arm carrying the *Ne1*-locus of *Triticumaestivum* L., Chinese Spring and the *Ne1*-expressivity. Wheat Inf. Serv..

[18] Lee J.H., Choi J.Y., Tao X.Y., Kim J.S., Kim W., Je Y.H. (2013). Transcriptome analysis of the small brownplanthopper, *Laodelphaxstriatellus* carrying rice stripe virus. Plant Pathol. J..

[19] Tang M., Mao D., Xu L., Li D., Song S., Chen C. (2014). Integrated analysis of miRNA and mRNA expression profiles in response to Cd exposure in rice seedlings. BMC Genomics.

[20] Kakumanu A., Ambavaram M.M.R., Klumas C., Krishnan A., Batlang U., Myers E., Grene R., Pereira A., Grene R. (2012). Effects of drought on gene expression in maize reproductive and leaf meristem tissue revealed by RNA-Seq. Plant Physiol..

[21] Wu Z.J., Li X.H., Liu Z.W., Xu Z.S., Zhuang J. (2014). *De novo* assembly and transcriptome characterization: Novel insights into catechins biosynthesis in *Camellia sinensis*. BMC Plant Biol..

[22] Wei W.L., Qi X.Q., Wang L.H., Zhang Y.X., Hua W., Li D.H., Lv H.X., Zhang X.R. (2011). Characterization of the sesame (*Sesamumindicum* L.) global transcriptome using Illumina paired-end sequencing and development of EST-SSR markers. BMC Genomics.

[23] Zou X.L., Tan X.Y., Hu C.W., Zeng L., Lu G.Y., Fu G.P., Cheng Y., Zhang X.K. (2013). The Transcriptome of *Brassica napus* L. Roots under water logging at the seedling stage. Int. J. Mol. Sci..

[24] Liu D.F., Sui S.Z., Ma J., Li Z.N., Guo Y.L., Luo D.P., Yang J.F., Li M.Y. (2014). Transcriptomicanalysis of flower development in winter sweet (*Chimonanthus praecox*). PLoS One.

[25] Shi X., Gupta S., Lindquist I.E., Cameron C.T., Mudge J., Rashotte A.M. (2013). Transcriptome Analysis of cytokinin response in tomato leaves. PLoS One.

[26] Cantu D., Pearce S.P., Distelfeld A., Christiansen M.W., Uauy C., Akhunov E., Fahima T., Dubcovsky J. (2011). Effect of the down-regulation of the high Grain Protein Content (GPC) genes on the wheat transcriptome during monocarpic senescence. BMC Genomics.

[27] Pont C., Murat F., Confolent C., Balzergue S., Salse J. (2011). RNA-Seqin grain unveils fate of neo- and paleopolyploidization events in bread wheat (*Triticumaestivum* L.). Genome Biol..

[28] Li A., Zhang R., Pan L., Tang L., Zhao G., Zhu M., Chu J., Sun X., Wei B., Zhang X. (2011). Transcriptome analysis of H_2_O_2_-treated wheat seedlings reveals a H_2_O_2_-responsive fatty acid desaturase gene participating in powdery mildew resistance. PLoS One.

[29] Oono Y., Kobayashi F., Kawahara Y., Yazawa T., Handa H., Itoh T., Matsumoto T. (2013). Characterisation of the wheat (*Triticumaestivum* L.) transcriptome by *de novo* assembly for the discovery of phosphate starvation-responsive genes: Gene expression in Pi-stressed wheat. BMC Genomics.

[30] Zhang S., Song G., Gao J., Li Y., Guo D., Fan Q., Sui X., Chu X., Huang C., Liu J. (2014). Transcriptome characterization and differential expression analysis of cold-responsive genes in young spikes of common wheat. J. Biotechnol..

[31] Mackey D., Belkhadir Y., Alonso J.M., Ecker J.R., Dangl J.L. (2003). *Arabidopsis RIN4* is a target of the type III virulence effector *AvrRpt2* and modulates RPS2-mediated resistance. Cell.

[32] Mackey D., Holt B.F., Wiig A., Dangl J.L. (2002). *RIN4* interacts with *Pseudomonas syringaetype* III effector molecules and is required for for *RPM1*-mediated disease resistance in *Arabidopsis*. Cell.

[33] Axtell M.J., Staskawicz B.J. (2003). Initiation of *RPS2*-specified disease resistance in *Arabidopsisis* coupled to the *AvrRpt2*-directed elimination of *RIN4*. Cell.

[34] Yang S., Hua J. (2004). A haplotype-specific Resistance gene regulated by BONZAI1 mediates temperature-dependent growth control in *Arabidopsis*. Plant Cell.

[35] Cheng S., Liu R., Gallie D.R. (2013). The unique evolution of the programmed cell death 4 protein in plants. BMC Evol. Biol..

[36] Baumann P., Cech T.R. (2001). Pot1, the putative telomere end binding protein in fission yeast and humans. Science.

[37] Shakirov E.V., Perroud P.F., Nelson A.D., Cannell M.E., Quatrano R.S., Shippen D.E. (2010). Protection of Telomeres 1 is required for telomere integrity in the moss *Physcomitrella patens*. Plant Cell.

[38] Gao Y., Zhang H., Gao Q., Wang L., Zhang F., Siva V.S., Zhou Z., Song L., Zhang S. (2013). Transcriptome analysis of artificial hybrid pufferfish Jiyan-1 and its parental species: Implications for pufferfish heterosis. PLoS One.

[39] Kornitzer D., Ciechanover A. (2000). Modes of regulation of ubiquitin-mediated protein degradation. J. Cell. Physiol..

[40] Zhang Y., Feng S., Chen F., Chen H., Wang J., McCall C., Xiong Y., Deng X.W. (2008). Arabidopsis DDB1-CUL4 ASSOCIATED FACTOR1 forms a nuclear E3 ubiquitin ligase with *DDB1* and *CUL4* that is involved in multiple plant developmental processes. Plant Cell.

[41] Park J.J., Yi J., Yoon J., Cho L.H., Ping J., Jeong H.J., Cho S.K., Kim W.T., An G. (2011). *OsPUB15*, an E3 ubiquitin ligase, functions to reduce cellular oxidative stress during seedling establishment. Plant J..

[42] Liu Z.B., Wang J.M., Yang F.X., Yang L., Yue Y.F., Xiang J.B., Gao M., Xiong F.J., Lv D., Wu X.J. (2014). A novel membrane-bound E3 ubiquitin ligase enhances the thermal resistance in plants. Plant Biotechnol. J..

[43] Liu Y., Wang F., Zhang H., He H., Ma L., Deng X.W. (2008). Functional characterization of the *Arabidopsis* ubiquitin-specific protease gene family reveals specific role and redundancy of individual members in development. Plant J..

[44] Zhu D., Bai X., Luo X., Chen Q., Cai H., Ji W., Zhu Y. (2013). Identification of wild soybean (*Glycine soja*) *TIFY* family genes and their expression profiling analysis under bicarbonate stress. Plant Cell Rep..

[45] Kumar P.G., Laloraya M., Wang C.Y., Ruan Q.G., Davoodi-Semiromi A., Kao K.J., She J.X. (2001). The autoimmune regulator (AIRE) is a DNA-binding protein. J. Biol. Chem..

[46] Pion S., Fontaine P., Desaulniers M., Jutras J., Filep J.G., Perreault C. (1997). On the mechanisms of immunodominance in cytotoxic T lymphocyte responses to minor histocompatibility antigens. Eur. J. Immunol..

[47] Defraia C.T., Wang Y., Yao J., Mou Z. (2013). Elongator subunit 3 positively regulates plant immunity through its histone acetyltransferase and radical *S*-adenosylmethionine domains. BMC Plant Biol..

[48] Smeds L., Künstner A. (2011). ConDeTri—A content dependent read trimmer for Illumina data. PLoS One.

[49] Trapnell C., Pachter L., Salzberg S.L. (2009). TopHat: Discovering splice junctions with RNA-Seq. Bioinformatics.

[50] Roberts A., Pimentel H., Trapnell C., Pachter L. (2011). Identification of novel transcripts in annotated genomesusing RNA-Seq. Bioinformatics.

